# Imaging Special Nuclear Material using a Handheld Dual Particle Imager

**DOI:** 10.1038/s41598-020-58857-z

**Published:** 2020-02-05

**Authors:** William M. Steinberger, Marc L. Ruch, Nathan Giha, Angela Di Fulvio, Peter Marleau, Shaun D. Clarke, Sara A. Pozzi

**Affiliations:** 1 University of Michigan, Department of Nuclear Engineering and Radiological Sciences, Ann Arbor, MI 48109 USA; 2 Los Alamos National Laboratory, Nuclear Engineering and Nonproliferation Division, Los Alamos, NM 87544 USA; 3 University of Illinois at Urbana-Champaign, Department of Nuclear, Plasma, and Radiological Engineering, Urbana, IL 61801 USA; 4 Radiation and Nuclear Detection Systems Division, Sandia National Laboratories, Livermore, CA 94551 USA

**Keywords:** Imaging techniques, Applied physics

## Abstract

A compact radiation imaging system capable of detecting, localizing, and characterizing special nuclear material (e.g. highly-enriched uranium, plutonium…) would be useful for national security missions involving inspection, emergency response, or war-fighters. Previously-designed radiation imaging systems have been large and bulky with significant portions of volume occupied by photomultiplier tubes (PMTs). The prototype imaging system presented here uses silicon photomultipliers (SiPMs) in place of PMTs because SiPMs are much more compact and operate at low power and voltage. The SiPMs are coupled to the ends of eight stilbene organic scintillators, which have an overall volume of 5.74 × 5.74 × 7.11 cm^3^. The prototype dual-particle imager’s capabilities were evaluated by performing measurements with a ^252^*Cf* source, a sphere of 4.5 kg of alpha-phase weapons-grade plutonium known as the BeRP ball, a 6 kg sphere of neptunium, and a canister of 3.4 kg of plutonium oxide (7% ^240^*Pu* and 93% ^239^*Pu*). These measurements demonstrate neutron spectroscopic capabilities, a neutron image resolution for a Watt spectrum of 9.65 ± 0.94° in the azimuthal direction and 22.59 ± 5.81° in the altitude direction, imaging of gamma rays using organic scintillators, and imaging of multiple sources in the same field of view.

## Introduction

A key property of special nuclear material (SNM) is that it emits neutrons and gamma rays, either passively or when actively interrogated. Neutrons do not make up a large portion of background radiation and are mostly produced from cosmic ray showers in the upper atmosphere^1,2^. Thus, detecting neutrons above a low and reasonably understood background can be a strong indicator of the presence of SNM.

**Figure 1:**
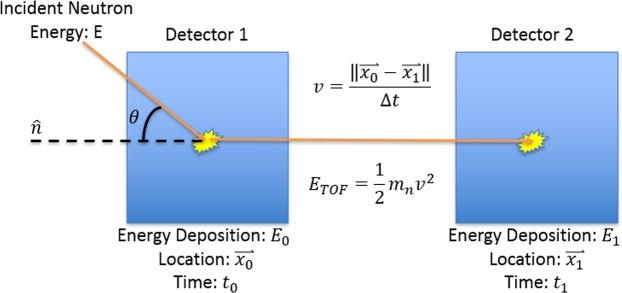
Depiction of a neutron double-scatter event.

Once a source is detected, it can be useful to discern the exact location of the source. Inspectors could use this capability to verify the location of a source in a safeguards scenario or emergency responders and war-fighters could use this capability to isolate a source. Several types of neutron imagers have been developed to accomplish this task, including coded-aperture imagers^3,4^, time-encoded imagers^5^, and neutron scatter cameras (NSCs)^6–12^. NSCs can be made significantly more compact and lighter than coded aperture or time-encoded imagers because no attenuating material or moving parts are necessary^13^. The primary characteristic that limits how compact a NSC can be made is the time resolution between coincident interactions^6^. NSCs function by using the subset of interactions in which a neutron scatters twice in the system between two independent or separated volumes. A depiction of a neutron double-scatter interaction is shown in Fig. 1. The scattering angle between the first interaction and the point of origin is derived by the kinematics of the multiple elastic scatters. If the time resolution is insufficient to resolve coincident interactions, then the sequencing of those interactions between the two independent volumes will have significant associated uncertainty with regards to which interaction happened first^14^. Incorrectly sequencing the interactions will cause the kinematic reconstruction to point in the wrong direction, ultimately producing artifacts in the images. The energy of the neutron after the first interaction is determined by the time-of-flight (TOF) between interactions, or *E*_*TOF*_. *E*_*TOF*_ is derived by the velocity as determined by the distance and time between the first and second interactions. Poor time resolution therefore, directly impacts the estimation of *E*_*TOF*_ leading to poor scattering angle reconstruction. Poor position resolution between interaction locations also reduces image quality by increasing uncertainty in *E*_*TOF*_, broadening cone pointing vectors and scattering angle projections in simple backprojection (SBP). The effects of poor position resolution and time resolution can be mitigated by increasing the distance between neutron interactions, but this change would increase the size of the imaging system, decrease the efficiency for double scatter interactions, and reduce the portability of such a system.

**Figure 2:**
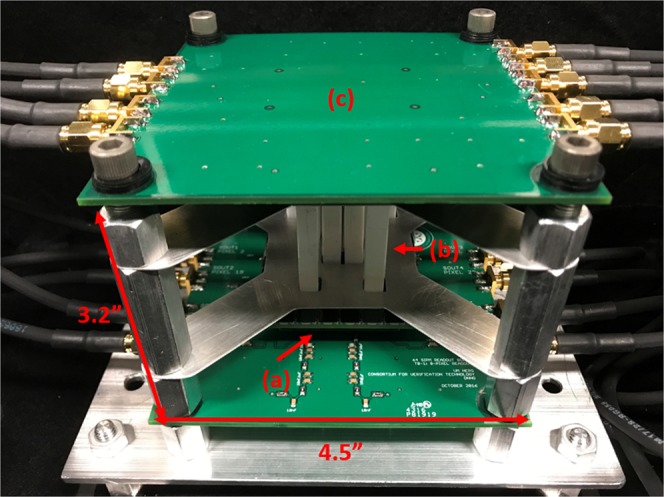
Photograph of the prototype handheld dual-particle imager composed of (**a**) two SensL C-Series SiPM arrays, (**b**) eight stilbene bars wrapped in polytetrafluoroethylene tape and (**c**) two custom printed circuit boards^35^.

Three aspects are required to create a compact NSC: small photo-detectors, sufficient time resolution between interactions and low-power photo-detectors so that a cooling system is not required. To meet the three stated requirements, substantial research was performed using stilbene organic scintillators coupled to silicon photomultipliers (SiPMs) to develop a NSC. Stilbene has high lightoutput relative to other hydrogenous scintillators, which could be used for the active volume of a NSC, and can achieve pulse shape discrimination (PSD) at lower energies compared to other hydrogenous scintillators^15,16^. SiPMs have recently become a viable choice as a photo-detector for scintillation light^17^. SiPMs also have advantages over traditional photomultiplier tubes (PMTs) in that they are not affected by magnetic fields and require low voltage/power to operate. Extensive research was performed to demonstrate that stilbene crystals coupled to SensL C-Series SiPMs have sufficient time resolution (280 ps standard deviation) and PSD capability to be used in a NSC^18,19^. In addition, previous work demonstrated that a position resolution of 4.9 mm along the length of a 6 × 6 × 50 *mm*^3^ bar of stilbene can be achieved by reading out both ends of the bar using SiPMs^20^. Encouraged from these measurements and guided by MCNPX-PoliMi simulations^21^, a prototype eight bar system was designed, constructed, and tested (Fig. 2). The following section details results from this prototype system, demonstrating neutron and gamma-ray imaging capability with in-lab sources and kilogram-quantities of SNM.

## Results

### Imaging neutrons

**Figure 3:**
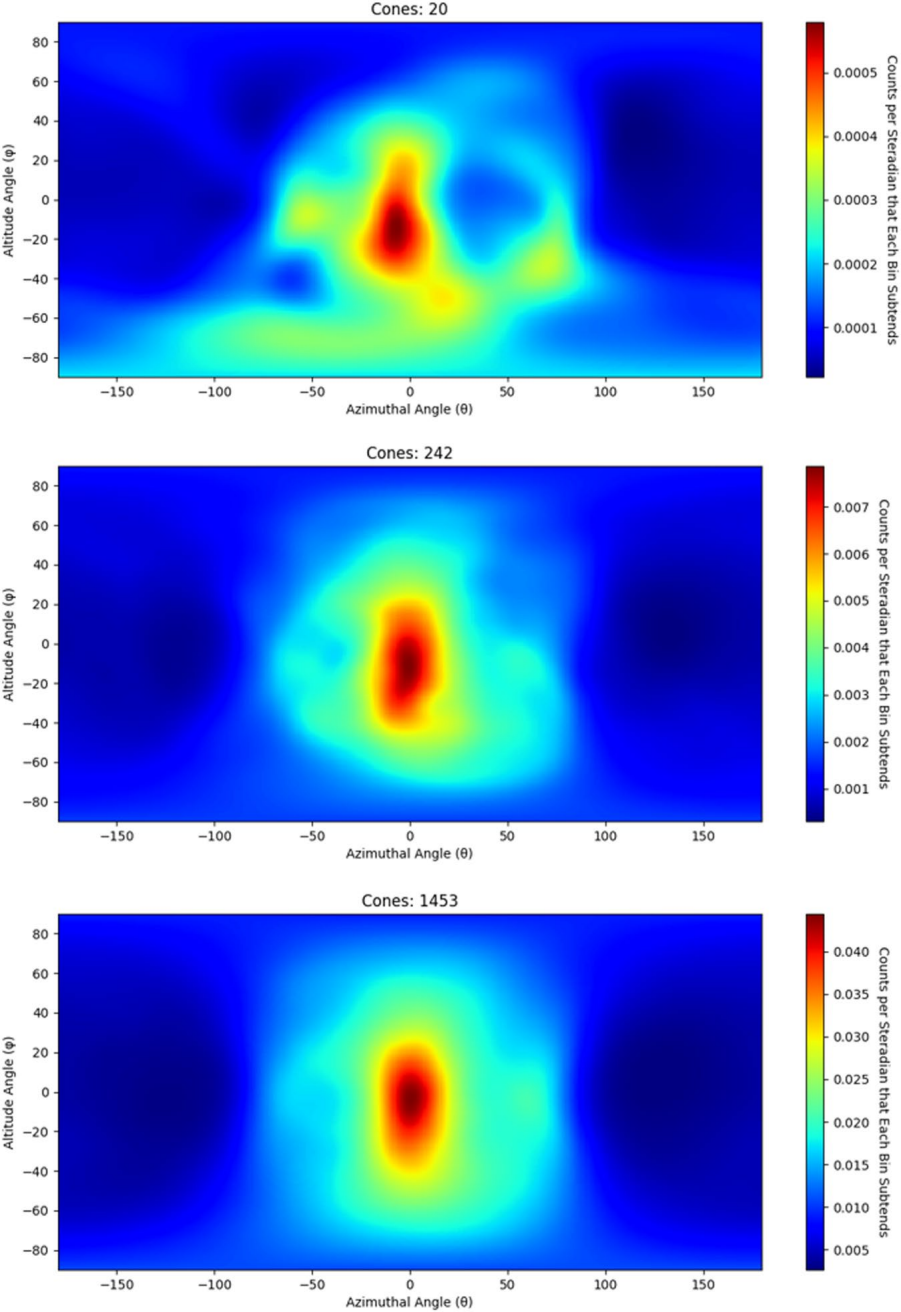
Neutron SBP images of a 1.2 × 10^7^ n/s ^252^*Cf* spontaneous fission source measured for 25 seconds (20 cones), 5 minutes (242 cones) and 30 minutes (1453 cones) at 58.4 cm from the center of the prototype H2DPI.

**Figure 4:**
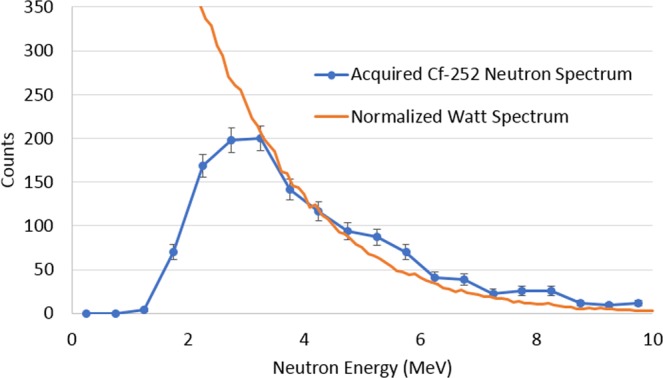
Acquired neutron energy spectrum from a 30 minute measurement of a 1.2 × 10^7^ *n*/*s* ^252^*Cf* spontaneous fission source at 58.4 cm from the center of the prototype H2DPI overlaid with a normalized Watt spectrum.

A 1.2 × 10^7^ *n*/*s* ^252^*Cf* spontaneous fission source was measured using the handheld dual particle imager (H2DPI) for 30 minutes with the source 58.4 cm from the center of the system. SBP images composed of 20, 252 and 1453 imageable events are shown in Fig. 3. These three images correspond to measurement times of 25 seconds, 5 minutes and 30 minutes demonstrating that the location of neutron sources can begin to be identified with a low number of imageable events. For an event to be imageable, the minimum requirement is that a neutron must scatter twice in the imager in two different bars. The light output from the first interaction is used to calculate the energy deposition by assuming the energy was deposited through elastic scattering off of hydrogen. The energy of the neutron after the first interaction, *E*_*TOF*_, is determined by the TOF of the neutron and the distance between interactions. The summation of these two energies yields the incident energy of the neutron. This feature makes the H2DPI a neutron spectrometer as well. Figure 4 shows the neutron spectrum from the image in Fig. 3. A normalized Watt spectrum, normalized to the measured value in terms of counts for the 3.25 mega-electron volts (*MeV*) energy bin, that is not efficiency-corrected is overlaid with the measured neutron energy spectrum and shows good agreement past 3 *MeV*. The reason for the insensitivity at lower energies is due to a 100 kilo-electron volts equivalent (*keVee*) light output threshold set on all interactions. 100 *keVee* corresponds to an energy deposition of 0.74 *MeV* because the conversion from light output to energy deposition is nonlinear for neutron interactions^22^. The conversion relationship for the stilbene pillars was measured in a time-of-flight experiment using a ^252^*Cf* in a similar experimental setup as described by Enqvist *et al*.^23^. Thus, the minimum energy neutron required to produce an imageable event would be just under 1.5 *MeV*. This threshold was chosen to reduce artifacts in the images produced by interactions with higher relative uncertainty. The following thresholds were also applied to the data: the minimum time difference between coincident neutron events is required to be greater than 250 *ps* and *E*_*TOF*_ has to be greater than the energy deposition in the second interaction. The first threshold of 250 *ps* is a single standard deviation of the measured time resolution of the system and is assumed to be constant for all coincident events. This threshold ensures that coincident neutron events are sequenced in the correct time order and sets an upper limit for *E*_*TOF*_ to be about 21.5 *MeV*. Approximately 5.6% of events that are classified as neutron double-scatter events by PSD thresholds are rejected due to this timing threshold. The other threshold is put in place to ensure physical events. A neutron cannot deposit more energy in the second interaction than its calculated *E*_*TOF*_.

**Figure 5:**
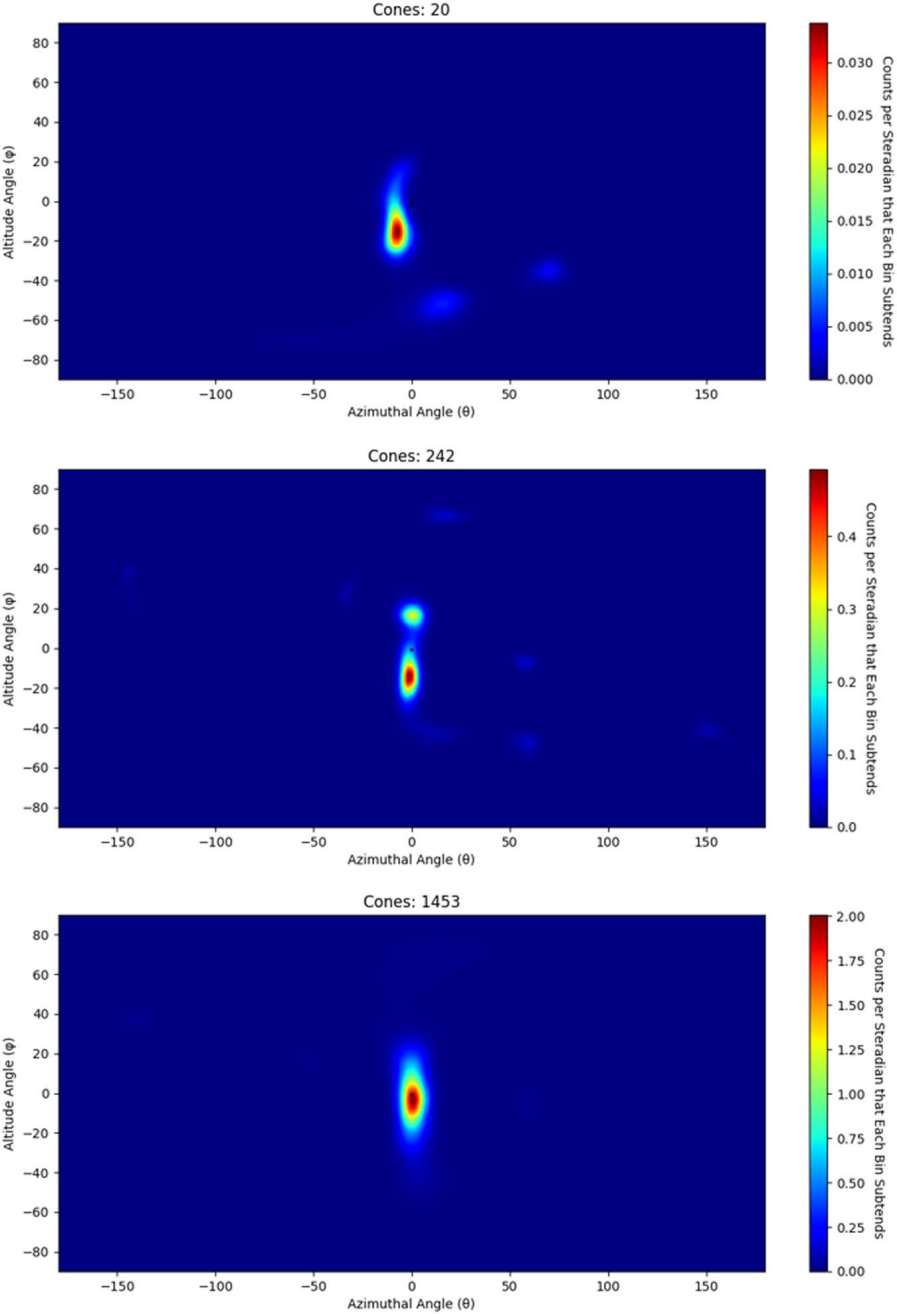
Neutron images of a 1.2 × 10^7^ n/s ^252^*Cf* spontaneous fission source measured for 25 seconds (20 cones with 23 iterations of LM-MLEM applied to the image), 5 minutes (242 cones with 50 iterations of LM-MLEM applied to the image) and 30 minutes (1453 cones with 27 iterations of LM-MLEM applied to the image) at 58.4 cm from the center of the prototype H2DPI. The source location is represented as a black dot.

To determine the most likely origin of neutrons from the measured source, list mode maximum likelihood expectation maximization (LM-MLEM) is applied to the image^24,25^. LM-MLEM is an iterative algorithm that converges on the most likely angular distribution from which a neutron originated. This analysis examined how non uniformly the images changed as a function of iteration value to determine a stopping criterion. (The method used is detailed in the Methods Section.) Each data set shown in Fig. 3 had LM-MLEM applied to produce the images shown in Fig. 5. The stopping criterion yielded an iteration value of 23 for the image containing 20 cones, 50 for the image containing 242 cones and 27 for the image containing 1453 cones.

To characterize the quality of the images from the prototype H2DPI, a data set consisting of 16,241 cones from a 6-hour measurement of a ^252^*Cf* source was analyzed using a bootstrapping technique. A random cone was sampled from the data set 1000 times and the following 1000 cones after that randomly sampled cone were analyzed to create an image. Image characteristics recorded included the location of the most likely pixel and full-width at half maximum (FWHM) in both the altitude and azimuthal directions. The average and standard deviation of the most likely pixel location was determined to be $(0.66\pm 0.56^\circ ,-\,1.82\pm 2.82^\circ )$. The FWHM in the azimuthal and altitude directions were determined to be 9.65 ± 0.94° and 22.59 ± 5.81° and are reported as the neutron image resolution of the system. The actual location of the ^252^*Cf* source was at 0° in the azimuthal direction and −0.8° in the altitude direction. The size of the source is assumed to be a point source since the mass of ^252^*Cf* is in the *μg* range. The actual locations are within a single standard deviation for the experimentally found altitude position and within 1.2 standard deviations for the azimuthal position.

**Figure 6:**
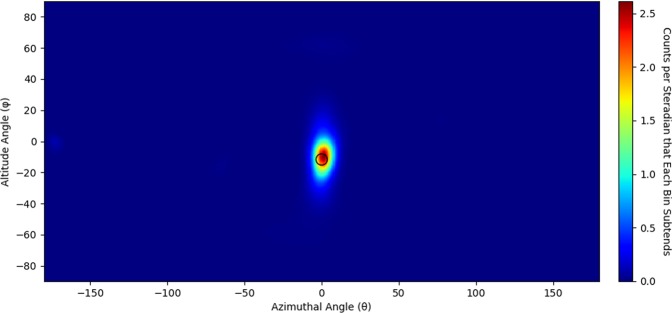
Neutron image of the BeRP ball (1660 cones with 27 iterations of LM-MLEM applied to the image). The approximate location and outline of the BeRP ball is shown in black.

In addition to measuring a ^252^*Cf* spontaneous fission source, a 15.8 hour measurement of a sphere of 4.5 kg of metal alpha-phase weapons-grade plutonium (WGPu) known as the BeRP ball was performed with the object approximately centered in the azimuthal direction and 58 cm from the center of the H2DPI. The neutron flux emitted by the BeRP ball was estimated to be 8.4 × 10^5^ *n*/*s*. This estimate was found by simulating the spontaneous fissioning of ^238^*Pu*, ^240^*Pu* and ^242^*Pu* in the BeRP ball using MCNPX-PoliMi^26^ given an initial isotopic concentration of the BeRP ball^27^. With the same thresholds used for the ^252^*Cf* analysis above, 1660 imageable events were analyzed to create a SBP image. LM-MLEM was applied to the data set of 1660 cones and the same stopping criterion used for the ^252^*Cf* source yielded an iteration value of 27 for the data set. Figure 6 shows the image of the BeRP ball with 27 iterations of LM-MLEM applied along with an outline of the BeRP ball that shows its approximate location.

### Imaging Gamma Rays

**Figure 7:**
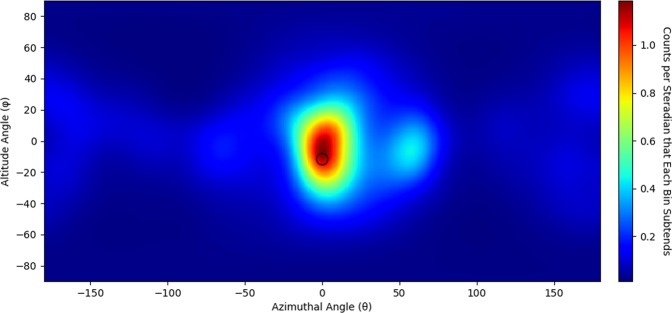
Gamma-ray image of the BeRP ball (12,352 cones with 41 iterations of LM-MLEM applied to the image). The approximate location and outline of the BeRP ball is shown in black.

Compton cameras determine the incident scattering angle of a gamma ray by analyzing the energy deposition of the first interaction and the total energy of the gamma ray. Traditional Compton cameras require the gamma ray to deposit a portion of its energy in the first interaction, and the rest in the second^28^, requiring the gamma ray to undergo photo-electric absorption in the second interaction. The dominant interaction mechanism for gamma rays in organic scintillators, however, is Compton scattering. Approximate Compton imaging is still possible with only organic scintillators even though the full energy of the gamma ray is not deposited^8^. Two adjustments must be made to image gamma rays using organic scintillators: the total energy of the gamma ray must be inferred based on the two interactions and the sequencing of the interactions must be determined. MCNPX-PoliMi^26^ was used to determine a correction factor to apply to the energy deposited. How this correction factor was found and applied is detailed in the Methods section. Only events with a time difference between two standard deviations of the time resolution, 0.5 ns, and three standard deviations of the time resolution, 0.75 ns were analyzed. The largest flight time of a gamma ray in the H2DPI is 0.27 ns over a flight path of 8 cm. Events occur within the defined window of 0.5–0.75 ns due to the time resolution of the system. Events could also occur due to chance coincidence, but we assume chance coincidence is negligible. We assumed that events within the defined time window can be correctly sequenced by timing. Applying these methods to the BeRP ball data set yields Fig. 7.

**Figure 8:**
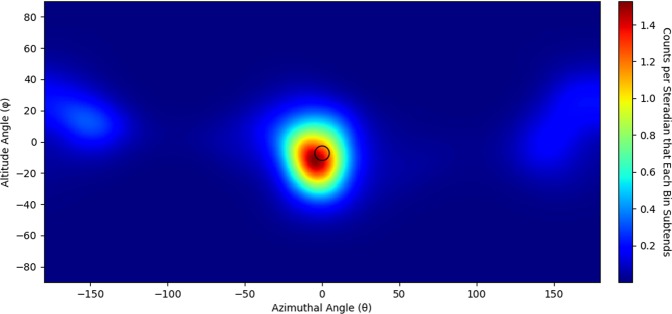
Gamma-ray image of a 6 kg sphere of neptunium (7441 cones with 180 iterations of LM-MLEM applied to the image). The approximate location and outline of the sphere of neptunium is shown in black.

An additional gamma-ray image was produced from a measurement of a 6 kg sphere of neptunium that was placed 55 cm away from the center of the H2DPI. The sphere is composed of mostly ^237^*Np*^29^ and emits 8700 *n*/*s*^30^, which is about 1% of the neutron intensity of the BeRP ball. We were not able to produce a neutron image of the sphere in the 45 minute acquisition time but were able to produce a gamma-ray image of the sphere as shown in Fig. 8.

### Imaging Multiple Sources

**Figure 9:**
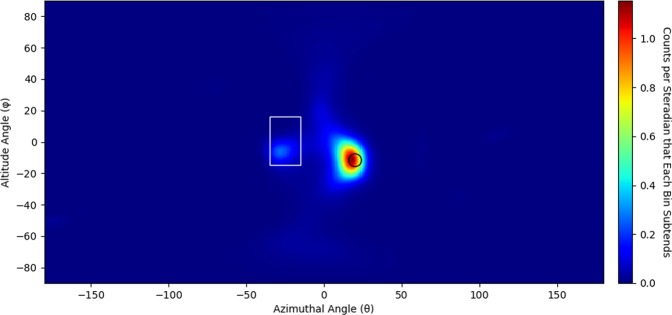
Neutron image of the BeRP ball (right) and a plutonium oxide canister (left) 57 cm away and separated by 50 cm (1283 cones with 31 iterations of LM-MLEM applied to the image). The approximate location and outline of the BeRP ball and the plutonium oxide canister is shown in black.

A 15.3 hour measurement of both the BeRP ball and a plutonium oxide canister (3.4 kg of 7% ^240^*Pu* and 93% ^239^*Pu* in oxide) was performed with the materials separated by 50 cm, and 57 cm away from the center of the H2DPI. This measurement acquired 1283 imageable events. Applying the convergence criterion detailed in the Methods section yielded a stopping iteration value of 31 iterations and is shown in Fig. 9. Two hot spots at (20, −10) and (−25, −5) show the BeRP ball and plutonium oxide canister respectively. An outline of the approximate location of the BeRP ball and the canister containing plutonium oxide are shown in black and white. An outline of the plutonium oxide within the canister is not shown since the exact geometry of the active volume of the plutonium oxide is not known and the exact source placement is not known.

## Discussion

National security missions require versatile detector systems to be able to detect SNM under a wide range of conditions, including complex shielding scenarios. In these cases, any information that can be acquired from a source can be useful to detect and localize it. For instance, being able to detect and image both gamma rays and fast neutrons allows for multiple methods to determine the location of a source of plutonium. This approach was demonstrated with the BeRP ball where the H2DPI was able to successfully produce neutron and gamma-ray images of the source. The ability to image both types of radiation is also beneficial for scenarios where only one type of particle may be present. The scenario detailed in this work is with ^237^*Np*, which does not emit a significant amount of neutrons relative to other spontaneous fission sources. We were not able to acquire any imageable neutron double scatter events over the 45 minute acquisition time. However, we were able to acquire 7441 imageable gamma-ray events to produce Fig. 8. Being able to image both neutrons and gamma rays can also help verify a source’s location. We were able to generate a gamma-ray image of the BeRP ball, giving two methods to image or verify the same source. Comparing the gamma-ray images to the neutron images, it can be seen that there are significantly more artifacts in the gamma-ray images. These artifacts are most likely due to a combination of incorrect sequencing and incorrect determination of the incident energy of the gamma ray. While resulting in more artifacts than are present in the neutron images, this ability to image gamma rays yields versatility in a compact imaging system.

Neutron images are presented for a ^252^*Cf* spontaneous fission source, the BeRP ball (4.5 kg of alpha-phase WGPu), and the BeRP ball along with a plutonium oxide canister in the same field of view. For the ^252^*Cf* point source, the image resolution was found to be 9.65 ± 0.94° in the azimuthal direction and 22.59 ± 5.81° in the altitude direction. Image characteristics from the BeRP ball did not significantly deviate from those measured with the ^252^*Cf* source. These results demonstrate that the H2DPI can image both sources in the laboratory and kilogram quantities of SNM. The image resolution was also validated by imaging multiple sources in the same field of view. The BeRP ball and a plutonium oxide canister were separated by 47° relative to the center of the H2DPI. Figure 9 shows the two sources separated by 45° with distinct space between the sources, meaning that both sources are resolved. The reason that the plutonium oxide source image is not as intense as the BeRP ball image is that the plutonium oxide yielded about half the number of incident neutrons on the H2DPI as the BeRP ball. LM-MLEM then converges on the most likely source distribution. Since it is more likely a neutron originates from the more intense BeRP ball, the image will converge more on that source. If the sources had the same neutron emission rate and spectrum, then the sources would converge with the same intensity^31,32^. Nevertheless, we were still able to resolve and image the two sources.

The prototype H2DPI presented was built to demonstrate that such a device made up of stilbene pillars coupled on both ends to SiPMs could image kilogram quantities of SNM. The above results prove this capability. Currently, the H2DPI is composed of only eight stilbene pillars with 56 unused SiPMs in each array. A 64-pillar system with the same footprint is a natural next step for a future deploy-able system. The intrinsic neutron double-scatter efficiency of such a system has been estimated to be 0.657%^21^, or about 66 times higher than the current intrinsic efficiency of the system. Such a system could acquire 20 imageable events from the BeRP ball at a distance of 2 meters in 64 seconds. Previous research with the prototype H2DPI has also shown that 20 cones can accurately reconstruct the source position with a confidence interval of 80%^33^.

In addition, future plans include the incorporation of inorganic scintillators into the H2DPI so that the system is composed of both stilbene and inorganic scintillators. Stilbene has relatively poor energy resolution compared to some inorganic scintillators such as *LaBr*_3_ or *CeBr*_3_. Incorporation of these types of scintillators would give users better spectroscopic information for source identification and allow for Compton imaging of gamma rays instead of the approximate method presented since inorganic scintillators have the density and atomic numbers necessary for photoelectric absorption of the gamma rays. These inorganic scintillators are not sensitive to fast neutrons and some optimization will have to be performed to determine how many inorganic scintillators should be incorporated and where the scintillators should be placed as to not significantly impact the neutron-imaging efficiency.

## Methods

A NSC works by detecting a neutron interaction in one detector and detecting the same neutron in another detector. The cosine squared of the angle, *θ*, at which the neutron scattered in the first interaction relative to the cone axis or “lever arm”, the vector between the two points of interaction^13^, is

1 $$\alpha =\,{\cos }\,{(\theta )}^{2}=\frac{{E}_{TOF}}{{E}_{TOF}+{E}_{0}}.$$

*E*_*TOF*_ is the energy of the neutron after the initial scatter. In 1, *E*_0_ is the energy deposited in the initial interaction. In

2 $${E}_{TOF}=\frac{1}{2}{m}_{n}{\left(\frac{\parallel {\overrightarrow{x}}_{1}-{\overrightarrow{x}}_{0}\parallel }{\Delta t}\right)}^{2},$$

${\overrightarrow{x}}_{0}$ and ${\overrightarrow{x}}_{1}$ are the first and second interaction locations and Δ*t* is the time difference between the two interactions. We used the method described by Ruch *et al*.^20^ to reconstruct the interaction location along the bar. Ratios that yielded positions outside the length of the bar were assigned positions at the end of the bar.

Once a scattering angle is determined for an event, the event must be projected on a sphere. To do this, a sphere of some radius is created and pixelated. Each pixel, *b*, has some position, ${\overrightarrow{x}}_{b}$, relative to the center of the detector. Determining a value for each pixel for a given cone, *d*, requires determining a Gaussian distribution for each interaction described in the following relation^13^:

3 $${C}_{d,b}={e}^{\frac{-{({\beta }_{b}-\alpha )}^{2}}{2\left({\sigma }_{\beta }^{2}+{\sigma }_{\alpha }^{2}\right)}}.$$

3 describes a Gaussian distribution with some effective mean denoted by *α* and all other points *β*_*b*_ with associated variance, *σ*^2^, for each. *β*_*b*_,

4 $${\beta }_{b}=cos{(\theta ^{\prime} )}^{2}=\frac{{(({\overrightarrow{x}}_{1}-{\overrightarrow{x}}_{0})\cdot ({\overrightarrow{x}}_{b}-{\overrightarrow{x}}_{0}))}^{2}}{|{\overrightarrow{x}}_{b}-{\overrightarrow{x}}_{0}{|}^{2}},$$

is equal to *cos*(*θ*′)^2^ where *θ*′ is the angle between the Cone Axis, $({\overrightarrow{x}}_{1}-{\overrightarrow{x}}_{0})$, and the vector between the initial interaction location and any pixel location on the projected sphere where the pixel location is denoted as ${\overrightarrow{x}}_{b}$. The variances for both *α* and *β*_*b*_ were determined for each location where uncertainties were propagated through the error propagation formula^34^:

5 $${\sigma }_{\alpha }^{2}={\left(\frac{\partial \alpha }{\partial {\overrightarrow{x}}_{0}}{\sigma }_{{\overrightarrow{x}}_{0}}\right)}^{2}+{\left(\frac{\partial \alpha }{\partial {\overrightarrow{x}}_{1}}{\sigma }_{{\overrightarrow{x}}_{1}}\right)}^{2}+{\left(\frac{\partial \alpha }{\partial \Delta t}{\sigma }_{\Delta t}\right)}^{2}+{\left(\frac{\partial \alpha }{\partial {E}_{0}}{\sigma }_{{E}_{0}}\right)}^{2}$$

and

6 $${\sigma }_{{\beta }_{b}}^{2}={\left(\frac{\partial {\beta }_{b}}{\partial {\overrightarrow{x}}_{0}}{\sigma }_{{\overrightarrow{x}}_{0}}\right)}^{2}+{\left(\frac{\partial {\beta }_{b}}{\partial {\overrightarrow{x}}_{1}}{\sigma }_{{\overrightarrow{x}}_{1}}\right)}^{2}.$$

For imaging gamma rays, the only difference is in the definition of *α*:

7 $$\alpha =cos{(\theta )}^{2}={\left(1+{m}_{e}{c}^{2}[\frac{1}{E}-\frac{1}{k\ast {E}_{2}}]\right)}^{2}\mathrm{}.$$

In Eq. 7, *m*_*e*_*c*^2^ is the rest mass of an electron, *E* is the energy of the incident gamma ray, *k* is the correction factor, and *E*_2_ is the energy deposited in the second interaction. *E* is defined as

8 $$E={E}_{1}+k\ast {E}_{2},$$

where *E*_1_ is the energy deposited by the gamma ray in the first interaction. The value of *k* was found using MCNPX-PoliMi. Mono-energetic gamma rays were simulated incident on the stilbene pillars with the arrangement shown in Fig. 2. The value of *k* was solved for double-scatter events by using Eq. 8 since *E* is known in simulation. It was found that the average value of *k* was 2.0 and the average value did not change significantly as a function of energy.

Once 3 is solved for every cone, the values are summed as described in

9 $${I}_{b}=\mathop{\sum }\limits_{d=1}^{D}\,{C}_{d,b}.$$

When these summed values are plotted such as what is shown in Fig. 3, a SBP image is created. Once a SBP image is created, LM-MLEM can be applied to the image. Mathematically, LM-MLEM is described in

10 $${\lambda }_{b}^{New}={\lambda }_{b}^{old}\,\mathop{\sum }\limits_{d=1}^{D}\,\frac{{n}_{d}^{\ast }{C}_{d,b}}{{\sum }_{b^{\prime} =1}^{B}\,{\lambda }_{b^{\prime} }^{old}{C}_{d,b^{\prime} }}.$$

In Eq. 10, ${\lambda }_{b}^{New}$ is the posterior source distribution, ${\lambda }_{b}^{old}$ is the prior source distribution and ${n}_{d}^{\ast }$ is the observation vector. The observation vector stores the number of times each observation type occurs within a data set. For LM-MLEM, each observation type occurs once unless you happen to get two identical events. Thus, for LM-MLEM, n* is just a vector of ones^13,24,25^. The summation from $d=1$ to *D*, for this application, refers to the summation over all cones and the summation from $b^{\prime} =1$ to *B* refers to the summation over all pixels in the image. To begin the iteration process, ${\lambda }_{b}^{old}$ is the initial SBP image. For further iterations, the posterior source distribution becomes the prior source distribution.

Once LM-MLEM is applied, convergence criterion must be applied to determine when to stop the iteration process. The method used for producing convergence criteria for the analysis above analyzed the variance in image difference from one iteration value to another to characterize how non-uniformly the image changed. In

11 $$Varianc{e}_{\Delta I}=Var({I}_{i+1}-{I}_{i}),$$

**Figure 10:**
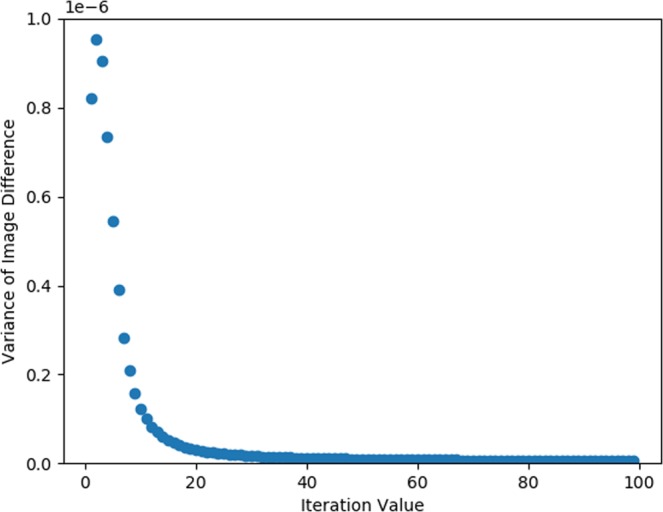
Variance in image difference as a function of iteration value.

*i* denotes the iteration value for LM-MLEM and *I* is the array of values making up the image for a given iteration value. Plotting this parameter, as a function of iteration value yields Fig. 10. The variance of the image difference changes significantly at low iteration values but begins to level off relative to the initial change around 30 iterations. The exact stopping criteria for neutron images was chosen to be the iteration value just under 2% the maximum value in the distribution seen in Fig. 10. The stopping criterion used to create the image in Fig. 5 made up of 20 cones, however, used a stopping criterion of 10%. Figure 10 was created with the data set of 1453 cones shown in Fig. 3. A stopping criterion of 10% was also chosen for the gamma-ray images.

## Data Availability

The datasets generated during and/or analyzed during the current study are available from the corresponding author on reasonable request.
